# Supplementary material for: Accounting for isoform expression increases power to identify genetic regulation of gene expression
Source: PLoS Comput Biol. 2024 Feb 12;20(2):e1011857. doi: 10.1371/journal.pcbi.1011857 (PMC10890775; doi:10.1371/journal.pcbi.1011857)
Supplement: S1 Text — Details on data preprocessing, software settings, and runtime analysis; quantile-quantile plot of GEUVADIS results; simulation results using genes with different numbers of isoforms. (PDF) [file pcbi.1011857.s001.pdf]

Supplementary Materials for: Accounting for Isoform Expression Increases Power to Identify Genetic Regulation of Gene Expression

Nathan LaPierre[1,5*], Harold Pimentel[2,3,4*]

1 Department of Computer Science, University of California, Los Angeles, California, United States

2 Department of Computational Medicine, University of California, Los Angeles, California, United States

3 Department of Human Genetics, University of California, Los Angeles, California, United States

4 Howard Hughes Medical Institute, Chevy Chase, Maryland, United States

5 Department of Human Genetics, University of Chicago, Illinois, United States

* nlapier2@uchicago.edu, hjp@g.ucla.edu

S1 Preprocessing of GEUVADIS data

We obtained genotype and RNA-Seq data from the GEUVADIS [1] dataset (see Code and data availability in the main text). For both our simulated dataset and real data analysis, we used the genotypes of 87 Yoruban donors in the GEUVADIS data. We used kallisto [2] version 0.46.0 to quantify the number of reads mapped to each isoform using the RNA-seq reads for the Yoruban samples, and discarded isoforms for which fewer than 50% of the samples had at least 5 reads mapped to that isoform.

For our real-data analysis, the transcripts per million (TPM) quantifications provided by kallisto were then normalized using the "DESeq2::estimateSizeFactorsForMatrix" function from the DESeq2 [3] R package. For our simulations, the original quantifications were discarded and new phenotype values were simulated according to the model presented in the main text.

S2 Preprocessing of GTEx data

We largely preprocessed the GTEx genotype and expression data in the way described in the Supplementary Materials for the GTEx v8 analysis [4]. The main differences were that i) we used only European samples to limit potential population stratification; ii) we performed low-expression filtering on isoform expression levels rather than aggregated gene expression levels; iii) normalization was applied to isoform expression levels rather than aggregated gene expression levels.

We obtained a GCT file containing isoform expression levels for the thyroid, a covariates file for the thyroid, and a population file listing the European samples from the GTEx portal website (see Code and data availability in the main text). We additionally obtained a VCF file containing the genotypes (Code and data availability). All files were subsetted to include only samples from the thyroid where covariates were available and the donor had European ancestry.

Genotypes were processed according to the GTEx v8 steps outlined in Section 2.3.4 of their Supplementary Materials [4]. Most of these steps apart from minor allele frequency (MAF) filtering at 1% had already been performed by the GTEx consortium prior to obtaining the VCF. The only difference in our pipeline is that we filtered out variants that were not genotyped in 100% of the samples. We used PLINK [5] v1.90b6.6 to perform the MAF and genotype call rate filtering. The resulting VCF had 10,489,762 SNPs compared to 10,726,114 reported in GTEx v8 [4].

Phenotypes were converted from GCT to BED format and annotated with the "Homo_sapiens.GRCh38.88.chr.gtf" file from Ensembl [6] (Code and data availability). We used this file instead of the GENCODE file used in the GTEx v8 analysis because the GENCODE file does not include isoform names, only gene names, whereas the Ensembl file does have the isoform names. The phenotypes were normalized with TMM [7], filtered for low expression, and then inverse-normal transformed as described in the GTEx v8 paper's Supplementary Material Section 3.4.2 [4]. Unlike in the GTEx v8 analysis, the phenotypes here were isoform expression levels, rather than gene expression levels.

S3 Software details

We wrote our own implementations of the Wilks-Bartlett test [8], the Cauchy aggregation test [9, 10], and the minimum p-value method. For Fisher's method [11], we used the version implemented in the scipy [12] python library. We used QTLtools [13] version 1.3.1. We wrote our own implementation of the FastQTL "Beta approximation" permutation scheme [14] and used this permutation scheme for all methods except QTLtools, which has its own implementation of this scheme built-in. For q-value false discovery rate (FDR) control [15], we used version 2.20.0 of the "qvalue" R package.

Any gene with a q-value of less than 0.1 (for simulations and GEUVADIS analysis) or 0.05 (for GTEx analysis) was considered an eGene identified by the method being evaluated. For GEUVADIS data and simulations, all methods were run with cis-windows of 50kb on each side of the gene's transcription start site. For GTEx, all methods were run with cis-windows of 1Mb on each side of the gene's transcription start site, to match the GTEx v8 analysis [4]. All methods were set to not output results or perform permutations on SNPs that had a nominal gene-level association p-value of greater than 0.5. In principle, all SNPs should be used to compute the p-values used in the permutation test described in the FastQTL paper [14]. However, in practice, this filtering step barely impacts results, since these SNPs have such low association signal, and results in a large speedup.

The Cauchy aggregation test [9] can in principle incorporate weighted p-values. Since the sample size is consistent across isoforms, we implemented the unweighted version of this statistic. It is possible that weights could be employed for some other purpose, e.g. if prior information is available, but this is outside the scope of the present study.

S4 Covariance simulation details

Most of the parameters of the simulation are described in the main text. Here, we describe how $\boldsymbol{\Phi}_j$ – the covariance matrix for the effects of a cis-SNP on isoform expression levels – and $\mathbf{V}$ and $\mathbf{W}$ – the covariance matrices used for simulation of the matrix normal noncis effects – are simulated. For all three, we start by generating a matrix of the appropriate dimension where the diagonal entries are 1 and the

off-diagonals are drawn from $Unif(\rho_{min}, \rho_{max})$, where ρ_{min} and ρ_{max} were set to 0.09 and 0.49 respectively for $\mathbf{\Phi}_j$ and $\mathbf{V}$, and were set to 0.99 each for $\mathbf{W}$.

This is not guaranteed to generate a positive semi-definite matrix, however. For $\mathbf{\Phi}_j$ we simply re-sample the matrix until it is positive semi-definite. For $\mathbf{W}$ this procedure is not tractable because the sample size is relatively large. Therefore, for $\mathbf{V}$ and $\mathbf{W}$, we sample a matrix $\mathbf{A}$ according to the procedure outlined above, then compute $\mathbf{A}^T\mathbf{A}$, which is guaranteed to be positive semi-definite. We then divided all entries in the resulting matrix by the maximum value in the matrix. We found that this generally resulted in diagonal values close to 1 and off-diagonal absolute values generally between 0.4 and 1 for $\mathbf{V}$ and generally between 0 and 0.2 for $\mathbf{W}$.

S5 Functional enrichment analysis details

We performed functional enrichment analysis using torus [16] and the SNP annotations provided in the GTEx v8 dataset [4] (available via the GTEx portal; see Code and data availability), mirroring the analysis performed in the GTEx v8 paper. We chose QTLtools-sum as a representative gene-level method because it is the most similar approach to FastQTL [14], which is used in the GTEx v8 analysis. We chose the F-test as a representative isoform-aware approach because it had the highest empirical power among methods that never had an inflated false positive rate in any simulation setting. We ran a "nominal" pass for each of these methods on the GTEx v8 data, in which all SNP-gene associations passing the standard $5 * 10^{-8}$ Bonferroni-corrected p-value threshold were called as eQTLs and input to torus; we only included SNP-gene pairs for genes that were identified as eGenes by each method.

Torus requires t-statistics for each SNP-gene association as part of its input. However, QTLtools-sum does not provide association statistics as part of its output and the F-test returns F statistics instead of t-statistics. Instead, we generated t-statistics from the p-values via

$$t = 1 - g(p/2, dof)$$

where t is the t-statistic we generate, g is the t-distribution CDF, p is the p-value,

and dof is the degrees of freedom, which we set to the sample size minus two. The sign of the statistic is not recoverable, but we found that flipping or randomizing the sign had no impact on the results.

S6 Running time analysis

The running times of the methods considered in the main text are difficult to compare precisely due to their different implementations and pipelines. We found that the running time mostly consists of running the SNP-isoform or SNP-gene regressions for each of the permutation replicates, rather than the specific testing approach employed. Accordingly, QTLtools [13], which implements the basic permutations and regressions very efficiently, ran much faster than the methods we implemented ourselves.

For example, we will examine the running time of the methods when run on GEUVADIS [1] chromosome 1. QTLtools-sum, QTLtools-mean, QTLtools-grpbest, and QTLtools-grppca1 all took 5.4-6.3 minutes to run. Running QTLtools on each isoform individually took 16.5 minutes, and then applying any of the p-value aggregation approaches on those results (e.g. "Fisher-QTLtools-iso" and so on) took less than 0.1 minutes each. The remaining approaches were bundled into a single script because they take longer and most of the running time consists of the basic regressions. This script took 777.8 minutes to run. Of this, SNP-isoform regressions took 391.3 minutes to run and the F-test (including multiple regression time) took 268.0 minutes to run. The time to run the remaining tests on the individual SNP-isoform regression results were as follows: 47.0 for Wilks-Bartlett, 39.3 for Fisher-perm, 2.3 for min-perm, and 7.8 for Cauchy-perm. The remaining approximately 20 minutes consisted of reading data, regressing out covariates, permuting isoforms, etc. Overall, the runtime clearly depends primarily on how efficiently the basic regressions are implemented, and the tests themselves take a relatively small amount of time. The Wilks-Bartlett and Fisher-perm methods, however, are slightly slower than Cauchy-perm and, obviously, min-perm.

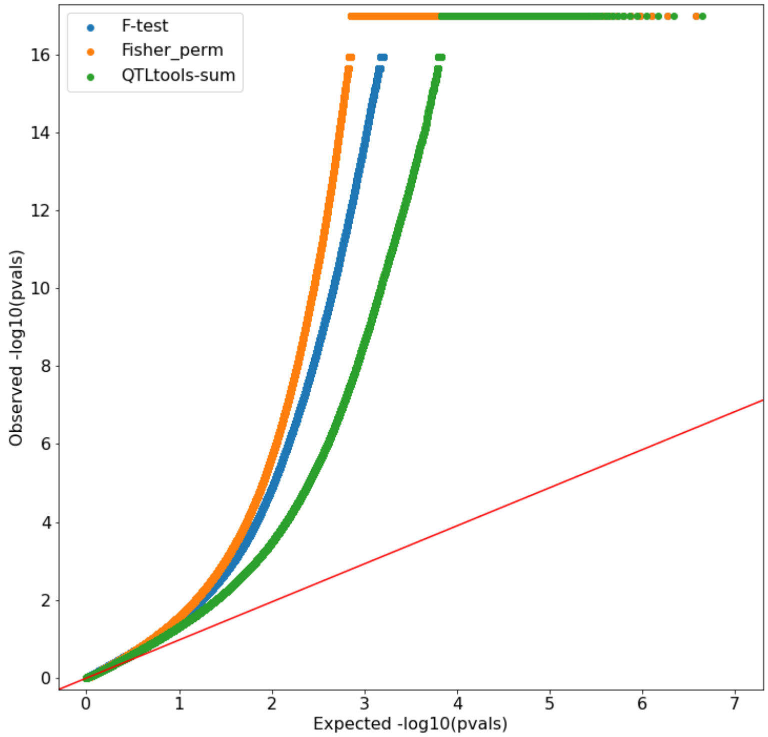

Fig A. Quantile-quantile (QQ) plot showing the expected distribution of all SNP-gene association p-values in the GEUVADIS data under the null (x-axis) versus the p-values empirically obtained from each method (y-axis). P-values that underflowed to 0 were manually set to 1e-17 for display purposes.

References

1. Lappalainen T, Sammeth M, Friedländer MR, 't Hoen PA, Monlong J, Rivas MA, Gonzalez-Porta M, Kurbatova N, Griebel T, Ferreira PG, Barann M. Transcriptome and genome sequencing uncovers functional variation in humans. Nature. 2013 Sep 26;501(7468):506-11.

2. Bray NL, Pimentel H, Melsted P, Pachter L. Near-optimal probabilistic RNA-seq quantification. Nature biotechnology. 2016 May;34(5):525-7.

3. Love MI, Huber W, Anders S. Moderated estimation of fold change and dispersion for RNA-seq data with DESeq2. Genome biology. 2014 Dec;15(12):1-21.

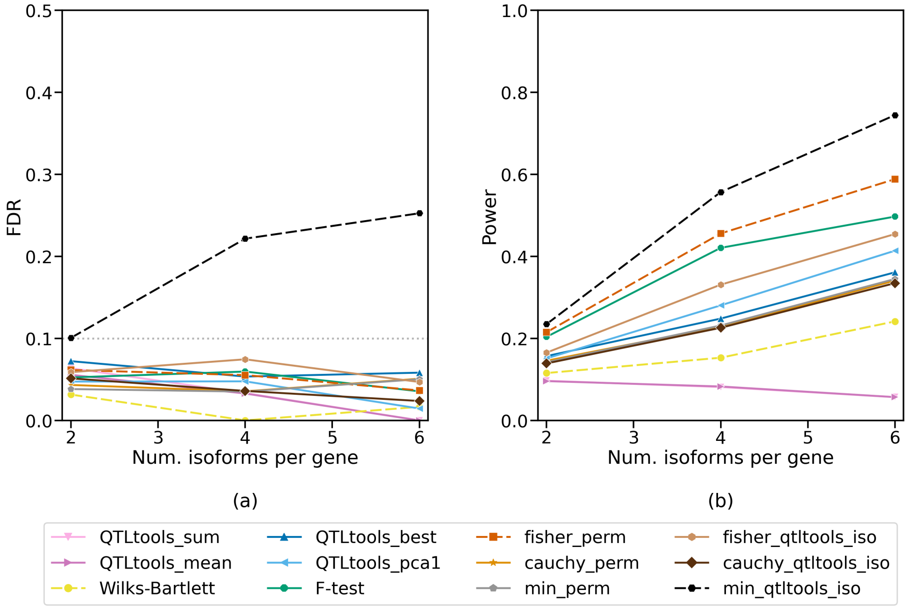

Fig B. Simulation results using genes with different numbers of isoforms. Genes with 4 isoforms were used to generate the main text results. In the main text results, genes with 4 isoforms were used. Number of isoforms is the X-axis, with empirical False Discovery Rate (FDR; left) or Power (right) on the Y-axis. Parameters were set to the "default" values listed in the main text. Dashed lines indicate methods which had inflated empirical FDR in at least one simulation setting. The method types are organized by color: Gene-level methods are in dark/light blue, QTLtools grouped permutation methods are in purple/pink, Wilks-Bartlett is in yellow, F-test is in green, and p-value aggregation methods are in oranges, browns, gray, and black.

4. GTEx Consortium. The GTEx Consortium atlas of genetic regulatory effects across human tissues. Science. 2020 Sep 11;369(6509):1318-30.

5. Purcell S, Neale B, Todd-Brown K, Thomas L, Ferreira MA, Bender D, Maller J, Sklar P, De Bakker PI, Daly MJ, Sham PC. PLINK: a tool set for whole-genome association and population-based linkage analyses. The American journal of human genetics. 2007 Sep 1;81(3):559-75.

6. Cunningham F, Amode MR, Barrell D, Beal K, Billis K, Brent S, Carvalho-Silva D, Clapham P, Coates G, Fitzgerald S, Gil L. Ensembl 2015. Nucleic acids research. 2015 Jan 28;43(D1):D662-9.

7. Robinson MD, Oshlack A. A scaling normalization method for differential expression analysis of RNA-seq data. Genome biology. 2010 Mar;11(3):1-9.

8. Johnson RA, Wichern DW. Applied multivariate statistical analysis. 6th ed. Upper Saddle River: Pearson Prentice Hall; 2007.

9. Liu Y, Xie J. Cauchy combination test: a powerful test with analytic p-value calculation under arbitrary dependency structures. Journal of the American Statistical Association. 2019 Apr 25.

10. Liu Y, Chen S, Li Z, Morrison AC, Boerwinkle E, Lin X. ACAT: a fast and powerful p value combination method for rare-variant analysis in sequencing studies. The American Journal of Human Genetics. 2019 Mar 7;104(3):410-21.

11. Fisher RA. Statistical methods for research workers. 1st ed. London: Oliver and Boyd; 1925.

12. Virtanen P, Gommers R, Oliphant TE, Haberland M, Reddy T, Cournapeau D, Burovski E, Peterson P, Weckesser W, Bright J, Van Der Walt SJ. SciPy 1.0: fundamental algorithms for scientific computing in Python. Nature methods. 2020 Mar;17(3):261-72.

13. Delaneau O, Ongen H, Brown AA, Fort A, Panousis NI, Dermitzakis ET. A complete tool set for molecular QTL discovery and analysis. Nature communications. 2017 May 18;8(1):15452.

14. Ongen H, Buil A, Brown AA, Dermitzakis ET, Delaneau O. Fast and efficient QTL mapper for thousands of molecular phenotypes. Bioinformatics. 2016 May 15;32(10):1479-85.

15. Storey JD, Tibshirani R. Statistical significance for genomewide studies. Proceedings of the National Academy of Sciences. 2003 Aug 5;100(16):9440-5.

16. Wen X. Molecular QTL discovery incorporating genomic annotations using Bayesian false discovery rate control. Ann App Stat. 2016 Sep;10(3):1619-1638.
