# Supplementary material for: Accounting for isoform expression increases power to identify genetic regulation of gene expression
Source: PLoS Comput Biol. 2024 Feb 12;20(2):e1011857. doi: 10.1371/journal.pcbi.1011857 (PMC10890775; doi:10.1371/journal.pcbi.1011857)
Supplement: Attachment — Submitted filename: IsoQTL_response_to_reviewers.pdf [file pcbi.1011857.s002.pdf]

January 15, 2024

Dear Editors of PLOS Computational Biology,

Thank you for considering our manuscript, “Accounting for Isoform Expression Increases Power to Identify Genetic Regulation of Gene Expression,” for publication in PLOS Computational Biology. We would like to thank the editors for their prompt handling of our manuscript and the reviewers for their constructive feedback. Following the reviewers’ suggestions, we have made several changes to the manuscript. We respond to the reviewer feedback point-by-point below. The original comments are in regular text, while our responses are in bold. Before getting to that, we briefly summarize the changes below.

- We have added a new supplementary text section on running time
- We have added two new supplementary plots: a QQ plot for the GEUVADIS results and a plot of the simulation results using genes with different numbers of isoforms (2/4/6)
- We have added simulation results using FeatureCounts as input data rather than Kallisto isoform quantifications
- Several of the reviewer comments, such as those about alternatives to the pipeline we evaluated (different FDR procedures, count-based data, quantifying exons rather than isoforms, etc) were addressed by adding text to the Discussion section
- We have made aesthetic improvements in Figure 2 (colors corresponding to method types, adjusted y-axis range)

Once again, we sincerely thank the reviewers for their constructive feedback, as we believe that the manuscript has become stronger as a result of addressing these points.

Best regards,

Harold Pimentel
Assistant Professor
University of California, Los Angeles & Howard Hughes Medical Institute

Reviewer #1: The authors detail four approaches to increasing detection power for regulatory variants affecting transcript abundance, using isoform level information. The paper is cleanly written and provides a useful reference for quantitative researchers.

We appreciate the Reviewer's kind words and feedback.

Major comments:

1. For the eGenes detected by isoform-aware methods and not by others, did these tend to have up/down patterns among the isoforms, such that gene-level signal would be masked through cancellation?

Indeed, we find that this is the case. Examining the eGenes with multiple isoforms found in GEUVADIS by the F-test approach and QTLtools-sum, we find that 88.3% (1221 of 1383) of such eGenes found by the F-test but not QTLtools-sum have isoforms with opposite-sign associations with the eQTL. Meanwhile, of the multi-isoform eGenes found by both methods, only 50.1% (326 of 651) have opposite-sign associations with the eQTL. If we require the opposite-sign isoform associations to be marginally significant at $p<0.05$, then 47.9% (663 of 1383) of the F-test-exclusive eGenes have significant opposite-sign associations, while only 22.1% (144 of 651) of eGenes found by both methods fit this criterion. We now note this in the relevant results section:

> **In general, we found that isoform-aware methods tended to find eGenes with isoforms that were genetically regulated in opposite directions, as expected. For example, we examined the eGenes with multiple isoforms identified by both the F-test and QTLtools-sum, versus those identified only by the F-test. We calculated the number of eGenes whose isoforms have opposite-sign associations with the eQTL, meaning that the eQTL had a positive-sign effect estimate on at least one isoform and a negative-sign estimate on at least one isoform. We found that 88.3% (1221 of 1383) of such eGenes found by the F-test but not QTLtools-sum have isoforms with opposite-sign associations with the eQTL. Meanwhile, of the multi-isoform eGenes found by both methods, only 50.1% (326 of 651) have opposite-sign associations with the eQTL. If we require the opposite-sign isoform associations to be marginally significant at $p<0.05$, then 47.9% (663 of 1383) of the F-test-exclusive eGenes have significant opposite-sign associations, while only 22.1% (144 of 651) of eGenes found by both methods fit this criterion.**

2. The authors treat isoform expression as observed variables in a matrix Y. Do the author have consideration or recommendation for working with count data? Additionally, is there any necessary consideration of the fact that counts are not observed from short read RNA-seq, but inferred from an upstream algorithm? Does performance in the simulation depend on the number of isoforms?

Incidentally, in response to Reviewer 2 comment 1, we ended up running QTLtools on GEUVADIS featureCounts data rather than gene expression levels quantified by kallisto. It seems that power was

maintained in this case – in fact, QTLtools found slightly more eGenes using featureCounts data than it did when run on summed kallisto isoform quantifications. The reviewer brings up a good point that quantification can pose challenges in some circumstances, particularly for lowly expressed genes or isoforms (e.g. [1]). In such cases it may be preferable to work with simpler count-based measures. Since these counts will be relatively low, it may be preferable to apply a Poisson or negative binomial model and run a generalized linear model rather than ordinary least squares. This is an interesting direction for future research. We have added the following text to the discussion section:

> **Another option is to work with read count data obtained from a method such as featureCounts or HTSeq, which could be either on the gene or exon level. We found in our analysis of GEUVADIS data that QTLtools run on featureCounts read counts obtained slightly more eGenes than QTLtools run on summed kallisto isoform quantifications, albeit still substantially fewer eGenes than isoform-aware methods. Reliably quantifying isoform expression can be challenging, particularly for lowly expressed isoforms. In such cases, it may be preferable to work with simpler count-based data such as that obtained from featureCounts. Since these counts will be relatively low, it may be preferable to apply a Poisson or negative binomial model and test for association using a generalized linear model rather than ordinary least squares. This is an interesting direction for future research.**

We also agree with the reviewer that it is important to show how the simulation results change as the number of isoforms per gene varies. We performed simulation experiments using genes with two or six isoforms rather than four. We show the trend in the plot below, which is now included as a supplementary figure. Broadly speaking, the difference between the isoform-aware and sum/average approaches grew as the number of isoforms increased. We explain this phenomenon with the following text, which has been added to the simulation results section:

> **Additionally, with negpct=0.5, as the number of isoforms per gene increased, the power of isoform-aware methods increased, while that of sum or mean approaches did not, or even slightly decreased (Supplementary Figure S2). This is likely due to the larger number of isoforms giving more opportunities for isoform-aware methods to identify true positive effects, while simultaneously creating more opportunities for these isoforms to “cancel out” when negatively correlated, harming sum or averaging approaches. We note, however, that when genes have many isoforms in real data, many of these isoforms may be lowly expressed, so this trend in our simulations may not translate precisely to real data.**

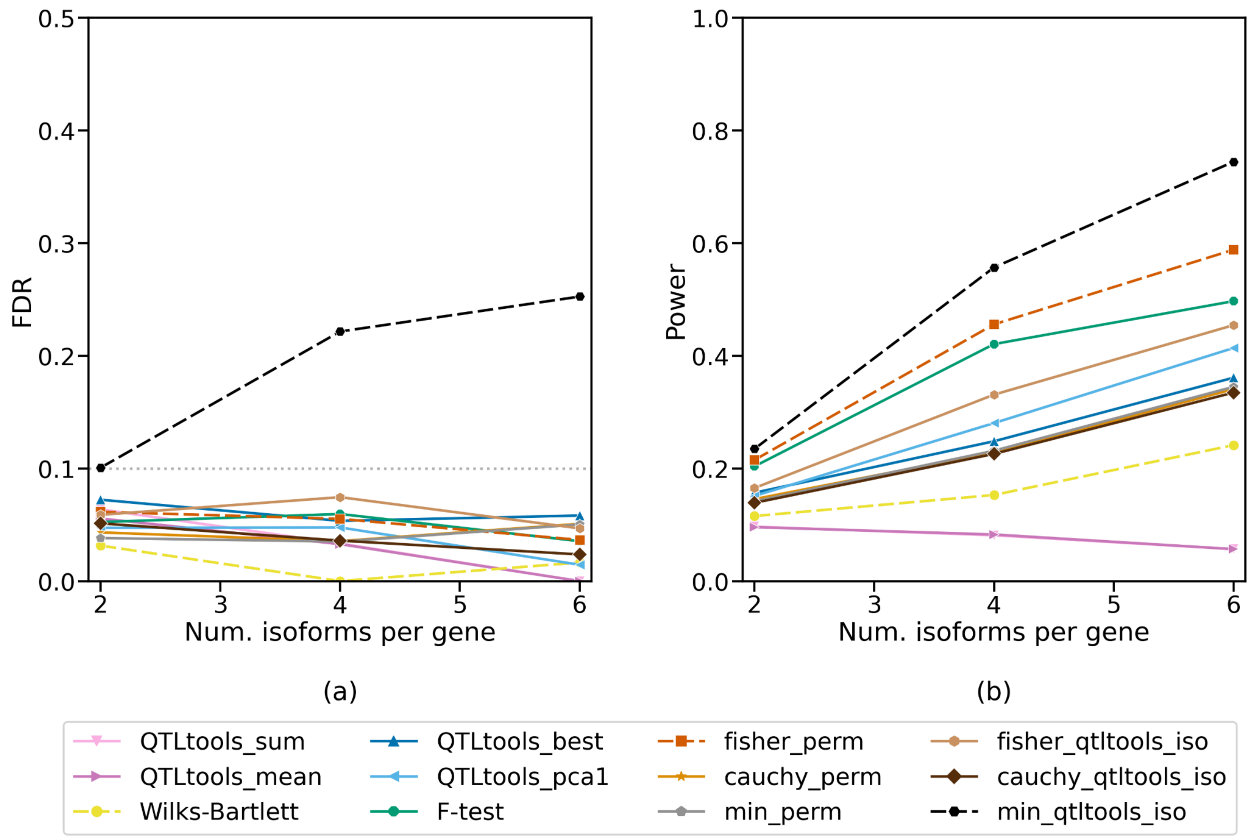

[1] https://bmcgenomics.biomedcentral.com/articles/10.1186/s12864-017-4002-1

3. The simulation mostly focuses on gene-level detection via rejection of null hypotheses. What about post-hoc identification of related isoforms, as in reference [15]? Which of methods are most easy to incorporate into a pipeline that also identifies affected isoforms, while controlling for error in the post-hoc step (again, as in reference [15]).

We thank the reviewer for bringing up this important point. It is true that the FastQTL-style FDR control approach that we use does not obviously admit a principled way to identify isoforms that drive the gene-level signal, beyond simply identifying individual isoform-level p-values that are significant. stageR (reference [15] in the original manuscript) suggests using the F-test or p-value aggregation in their "screening stage". We think the Wilks-Bartlett test could also be easily adapted to their framework. It seems unlikely that the QTLtools "best permutation" scheme could easily be adapted, since it does not first produce an aggregated p-value before subjecting it to global FDR control. We chose to focus on the FastQTL approach to FDR control for simplicity and because it seemed effective, but it is true that identifying driver isoforms may also be of interest. We think comparisons between these approaches is an interesting direction for future work. We have updated our discussion section to reflect this; see below.

> **Another dimension in which this problem can be approached differently is in the choice of FDR control. In this manuscript we used the beta-distributed approximate permutation test (Methods) along with Storey's Q-value, as suggested by FastQTL, to control FDR. Alternative approaches include the overall FDR and hierarchical FDR. We found the FastQTL approach to be effective for controlling FDR, and for simplicity, we do not evaluate these alternatives here. However, one disadvantage of this approach is that it focuses on gene-level testing, and does not obviously suggest a principled way to identify the isoforms that drove the gene-level signal (apart from identifying individual isoform-level p-values that are significant). Methods such as stageR offer an alternative approach to FDR control that does allow for identifying the driver isoforms. In the stageR method, approaches that we evaluated in this manuscript such as the F-test or p-value aggregation form an initial ``screening stage,'' which is then followed by a ``confirmation stage'' in which the individual (isoform-level) tests are re-assessed and controlled for FDR or Family-Wise Error Rate. It would be interesting to compare the stageR and FastQTL (and other) approaches in future work.**

4. Often genetic variants modifying expression level of genes are also of interest as mediators for downstream traits, as the authors note in the introduction, "Many loci identified in GWAS lie in non-coding regions of the genome, so their functional role is not immediately clear, though it is expected that many such loci play a role in gene regulation Molecular Quantitative Trait Loci (QTL) mapping studies help address this challenge." How may isoform expression be implicated as mediators of downstream GWAS traits? What considerations from this work may extend to the subsequent question of involvement in GWAS traits.

We thank the reviewer for bringing up this important consideration. Indeed, a great recent example of this has just been published, showing that isoform-level modeling increases power in Transcriptome-Wide Association Studies (https://doi.org/10.1038/s41588-023-01560-2), and we think it is important to make a note of that in our Discussion. We have added the following text:

> **Finally, similar approaches may be used to more-powerfully identify expression or other molecular traits that mediate genetic effects on complex traits and diseases. Indeed, recent work has shown that isoform-level modeling increases power in Transcriptome-Wide Association Studies (TWAS).**

Minor comments:

1. Figure 2, it would be useful to distinguish one way or another the methods presented here according to the four major groupings presented in the main text: traditional, grouped permutation tests, general linear model, multiple regression. In the caption the authors have Wilks-Bartlett, QTLtools sum, QTLtools best, QTLtools pca1, QTLtools mean, F-test, fisher perm, min perm, cauchy perm, fisher qtltools iso, min

qtltools iso, cauchy qtltools iso. While these can be associated with the groupings by cross-referencing, it would be beneficial to make this explicit in naming of color groups.

We appreciate this suggestion and have grouped the methods by color – see updated Figure 2 and its caption (which now explains this grouping). As noted in the updated caption,

> **The method types are organized by color: Gene-level methods are in dark/light blue, QTLtools grouped permutation methods are in purple/pink, Wilks-Bartlett is in yellow, F-test is in green, and p-value aggregation methods are in oranges, browns, gray, and black.**

Reviewer #2: This manuscript by LaPierre and Pimentel examines several statistical and computational approaches to identify genes that have at least one isoform whose expression level is regulated by genetic variants. While the problem of identified eGenes is not new this manuscript explains very well the issue that most genes have multiple isoforms and that correctly identifying eGenes is still an open problem. This is an important issue as eGenes may be important for gaining a better understanding of the molecular mechanisms underlying the connection between genetic variants and human traits. The simulations and the two real datasets provide an excellent setting to compare the approaches and understand their advantages and limitations. While some of the approaches are not new, their application for this problem and comparison have not been studied before. Bringing attention to account for different gene isoforms is timely and needed.

We appreciate the Reviewer's kind words and feedback.

I have some relatively minor comments and suggestions to improve the manuscript.

#1) While it is understandable that one needs Kallisto/Salmon isoform level quantification for answering the specific question the authors frame for this paper. In many cases the quantification at the gene level could be done in a simpler way by just aligning the reads with a tool like Hisat2/STAR and then just use a simplified gene model to obtain the read count for the gene. An approach like this should be discussed for context as this has been traditionally done. I understand this may be similar to the QTLtool-sum/mean but it is not exactly the same.

We agree that it is worthwhile to mention this option. Additionally, we felt that it would be interesting to compare how this does versus the "QTLtools-sum" approach. Applying QTLtools+featureCounts (QTLtools run on featureCounts gene read counts) to the GEUVADIS data, we found that QTLtools+featureCounts identified more eGenes than QTLtools-sum, albeit still substantially fewer than all isoform-aware methods. We have made a note of this in the results section:

> **A common alternative to summing isoform-level quantifications as we did for QTLtools-sum is to use read counting or alignment methods such as featureCounts, HTSeq, or STAR to quantify gene expression levels. We also ran QTLtools on gene read counts obtained from featureCounts. This approach identified more eGenes compared to the QTLtools-sum approach (1718 versus 1552) but substantially fewer than the isoform-aware methods, which each found 2342 or more eGenes.**

#2) Related to #1,How does the eGENE summary data compare to the summary data from the results consortia provided themselves?

We identified substantially more eGenes in the Yoruban population than in the original GEUVADIS study, including the “gene-level” approaches we tested. Our gene-level approaches identified 1294-1553 eGenes, while the original GEUVADIS study identified 501. We attribute this difference to several substantial differences in analysis pipelines, in part due to evolving practices in RNA-seq data analysis since 2013. We have added the following text:

> **We note that the original GEUVADIS study identified 501 eGenes in the Yoruban samples. We attribute this difference to several factors in the analysis pipeline, including our use of kallisto and DESeq size factors to quantify expression levels versus the original study's use of summed transcript reads per kilobase million (RPKM, which is now recommended against in current practice) and the original study's use of Matrix eQTL to call eGenes.**

As for GTEx, we note in section 2.4 that the equivalent approach (QTLtools-mean) to the one used in GTEx (FastQTL) results in fewer eGenes than in the original consortium:

> **However, QTLtools-mean found fewer eGenes than the GTEx v8 eQTL analysis on the thyroid (13477), which was expected given the differences in the analysis pipeline described above.**

And we list these differences as follows:

> **The main differences were that i) we used only European samples to avoid potential population stratification; ii) we performed low-expression filtering on isoform expression levels rather than aggregated gene expression levels; iii) normalization was applied to isoform expression levels rather than aggregated gene expression levels (Supplementary Note).**

We believe that these differences explain the discrepancy.

#3) There should also be some discussion on approaches that may map reads to exons first and then QTL map the individual exons rather than the isoforms. This likely will not solve the correlation issues as exons are linked together, but it may deserve some discussion.

We thank the reviewer for bringing up this consideration. We have added the following text to the Discussion, reflecting this.

> **A similar approach that we do not consider here is using exon quantifications rather than isoform quantifications, which can also be computed using kallisto or Salmon. Alternatively, differential exon usage methods like DEXSeq could be adapted for eQTL/eGene analysis. While many reads will be compatible with multiple isoforms, presenting a challenge for estimating individual isoform quantities, this is generally less of an issue for exon quantification. While this is possible, it has been noted that these methods suffer from other issues such as not utilizing full information of junction reads.**

#4) It is a bit surprising that permutation after Fisher aggregation (Fisher-perm) is sensitive to the higher level of \sigma^2_h. I would have expected that the permutation would correct the p-values accordingly. Could there be an issue on how the permutation is done?

We agree with the reviewer that this deserves careful attention. We think it is unlikely that we have implemented the permutation scheme incorrectly, as we carefully compared it to the original implementation in the FastQTL package to ensure that the results agreed. In the original text, we refer to Poole et al (https://doi.org/10.1093/bioinformatics/btw438) for a discussion on how Fisher's method yields too-strong p-values when applied to dependent p-values (as is well-known). Poole et al. also briefly discuss how FDR control with Q-values can be miscalibrated for Fisher's method due to the difficulty in fitting the null distribution (as you alluded to in a subsequent comment). However, it is a little bit trickier to diagnose in this case because, as you note, the input to the Q-value method in this case is the Beta-distributed approximate permutation p-values.

We performed some additional simulation experiments using genes from GEUVADIS chromosome 22 to see if we observed similar results when increasing the number of permutations used to set the Beta distribution parameters from 100 to 1000, or using the direct permutation p-values rather than the Beta approximated p-values as input to the Q-value method. We consistently observed that Fisher-perm had an increasingly inflated empirical FDR as sigma^2_h increased, and that min-perm and Cauchy-perm did not, in all of these cases.

It is possible that the Beta approximation is not suitable in cases where the individual p-values are badly miscalibrated, and/or that an exhaustive permutation approach with very many permutations (which is not computationally feasible in this case) would lead to Fisher's method being calibrated.

#5) It would be useful to see the QQplots for the real datasets results, and perhaps to calculate genomic inflation factors, or compare observed p-value values vs what would be expected at p=0.1 across the multiple methods. The shape of the histogram tail can also influence Storey's q-value method and may also be interesting to look at that.

We agree with the reviewer that this is interesting to examine. We have generated a QQ plot for the GEUVADIS data, shown below and now included in the Supplementary document. All SNP-gene p-values across the genome (for all SNPs in cis for each gene) are shown. All methods differ substantially from the null distribution of p-values, which is expected as the null hypothesis here is that no SNPs genetically regulate any genes. We note, however, that most of the points on the plot are located in the 0-2 range on the x- and y-axes, which is hard to see visually since there are several million points on the plot. As the reviewer suggests, it is instructive to compute the ratio of the -log10(p-values) at the 90th percentile (or 10th percentile of non-transformed p-values) for each method compared to that of the expected distribution. These ratios are: 1.49 for F-test, 1.57 for Fisher-perm, and 1.31 for QTLtools-sum. Traditional genomic inflation factors (i.e. based on test statistics) cannot be easily computed here because the different methods use different test statistics.

We are more familiar with the use of QQ plots in GWAS studies or to test whether eQTL variants are enriched for GWAS signal relative to all GWAS variants. Thus, we searched the literature to try to find other studies that have pooled SNP-gene associations for large-scale eQTL studies into a single QQ plot. We found two such studies, Sieberts et al 2020 [1] (see Figure 1a) and Gao et al 2014 (see Supplementary Figure S13). The QQ plot of Sieberts et al is considerably more inflated than ours, while the QQ plot of Gao et al is less inflated but still quite inflated at the extreme. This gave us some reassurance that our results are reasonable.

[1] Sieberts, Solveig K., et al. "Large eQTL meta-analysis reveals differing patterns between cerebral cortical and cerebellar brain regions." Scientific data 7.1 (2020): 340.

[2] Gao, Chuan, et al. "HEFT: eQTL analysis of many thousands of expressed genes while simultaneously controlling for hidden factors." Bioinformatics 30.3 (2014): 369-376.

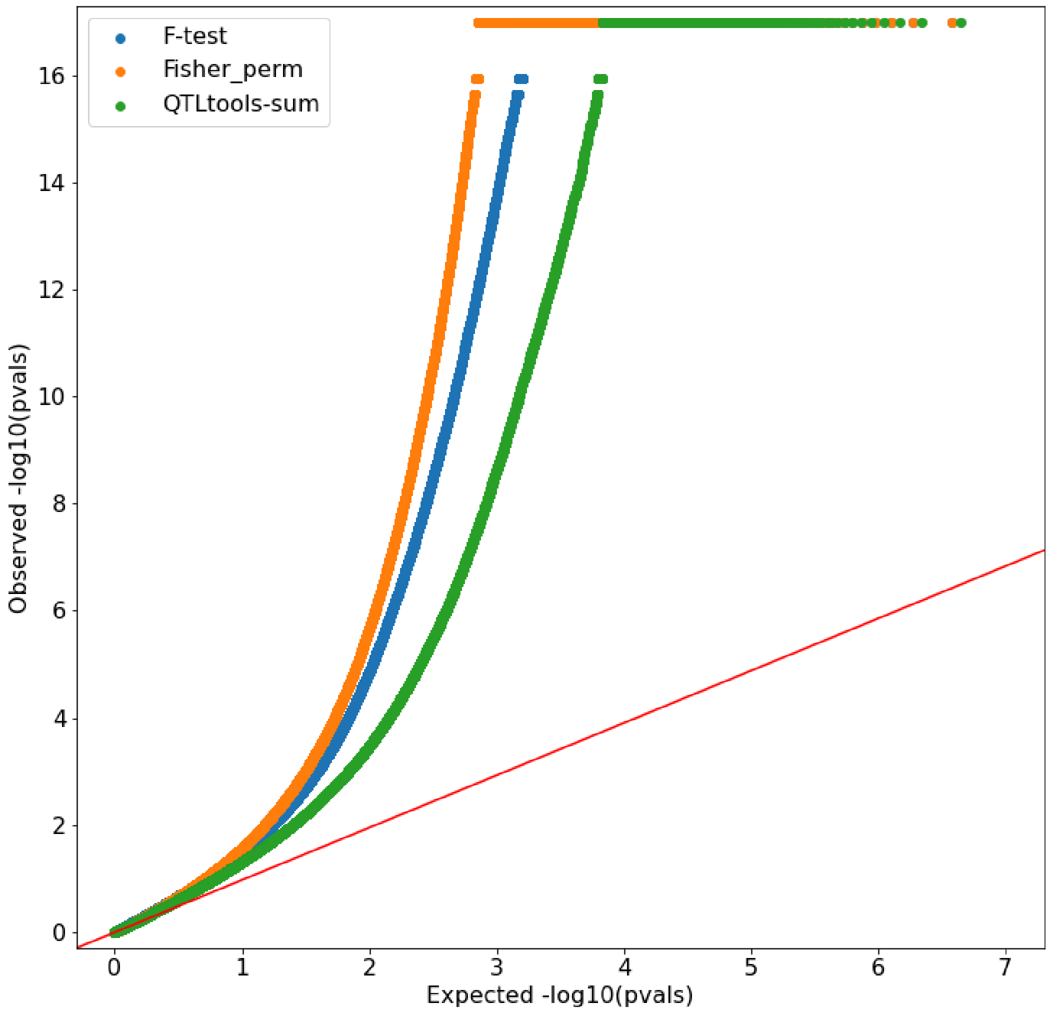

#6) It would be useful to also provide an estimate of the computational complexity of the approaches. Which approaches would take more time if they require more permutations or more complex computations?

We agree with the reviewer that this is an important consideration. The running times of the methods are difficult to compare precisely due to their different implementations and pipelines. We found that the running time mostly consists of running the SNP-isoform or SNP-gene regressions for each of the permutation replicates, rather than the specific testing approach employed. Accordingly, QTLtools, which implements the basic permutations and regressions very efficiently, ran much faster than the methods we implemented ourselves.

For example, we will examine the runtime of the methods on GEUVADIS chromosome 1. QTLtools-sum, QTLtools-mean, QTLtools-grpbest, and QTLtools-grppca1 all took 5.4-6.3 minutes to run. Running QTLtools on each isoform individually took 16.5 minutes, and then applying any of the p-value aggregation approaches on those results (e.g. "Fisher-QTLtools-iso" and so on) took less than 0.1 minutes each. The remaining approaches were bundled into a single script because they take longer and most of the runtime consists of the basic regressions. This script took 777.8 minutes to run. Of this, SNP-isoform regressions took 391.3 minutes to run and the F-test (including multiple regression time) took 268.0 minutes to run. The time to run the remaining tests on the individual SNP-isoform regression results were as follows: 47.0 for Wilks-Bartlett, 39.3 for Fisher-perm, 2.3 for min-perm, and 7.8 for Cauchy-perm. The remaining approximately 20 minutes consisted of reading data, regressing out covariates, permuting isoforms, etc. Overall, the runtime clearly depends primarily on how efficiently the basic regressions are implemented, and the tests themselves take a relatively small amount of time. The Wilks-Bartlett and Fisher-perm methods, however, are slightly slower than Cauchy-perm and, obviously, min-perm.

We have added a section on running time to the supplementary note, with text very similar to that which we gave here.

#7) Figure 2, it would be useful to zoom in the FDR y-axis, and do not have that much white space.

We appreciate the reviewer's suggestion and have zoomed in the FDR y-axis so that its maximum is 0.5 – please see the updated Figure 2. While the left two plots could have been zoomed in further, we left the maximum at 0.5 in order to have it consistent across the three FDR plots for the sake of easy visual comparison.
